# Confirm RX™ Cardiac Monitor Placement in a Pediatric Patient

**DOI:** 10.19102/icrm.2018.090912

**Published:** 2018-09-15

**Authors:** Orhan U. Kilinc, Jill K. L. Shivapour, Christopher S. Snyder

**Affiliations:** ^1^Division of Pediatric Cardiology, Department of Pediatrics, Rainbow Babies and Children’s Hospital, Cleveland, OH, USA

**Keywords:** Cardiac monitor, loop recorder, pediatric

## Abstract

We present the first known report of a pediatric implantation of the Bluetooth™-enabled Confirm RX™ insertable cardiac monitor (Abbott Laboratories, Chicago, IL, USA) in a 17-year-old patient with unexplained syncopal episodes. This case illustrates the ability to obtain immediate rhythm information from a patient using a Bluetooth™-enabled device following a minimally invasive procedure.

## Case presentation

We report the first, to our knowledge, pediatric implantation of the Bluetooth™-enabled Confirm RX™ insertable cardiac monitor (Abbott Laboratories, Chicago, IL, USA) in a pediatric patient.

The patient was a 17-year-old male with a one-year history of unexplained syncopal episodes. The episodes were reported to last five minutes and to have occurred in multiple settings (eg, riding a bike, talking, standing, driving a fence post into the ground), and the patient denied any prodrome. Family history was remarkable for uncontrolled seizures (mother and two maternal aunts), sudden infant death syndrome (another maternal aunt), and drowning (distant maternal relative). The patient’s physical examination was normal, as were his electrocardiograms, exercise stress test, 14-day heart rhythm monitor, and procainamide challenge results.^1^ After discussing the benefits, limitations, and complications of an insertable cardiac monitor with the patient and family, informed consent was obtained and the patient was brought to the electrophysiology laboratory for the implantation.

## Procedure

**Figure 1:**
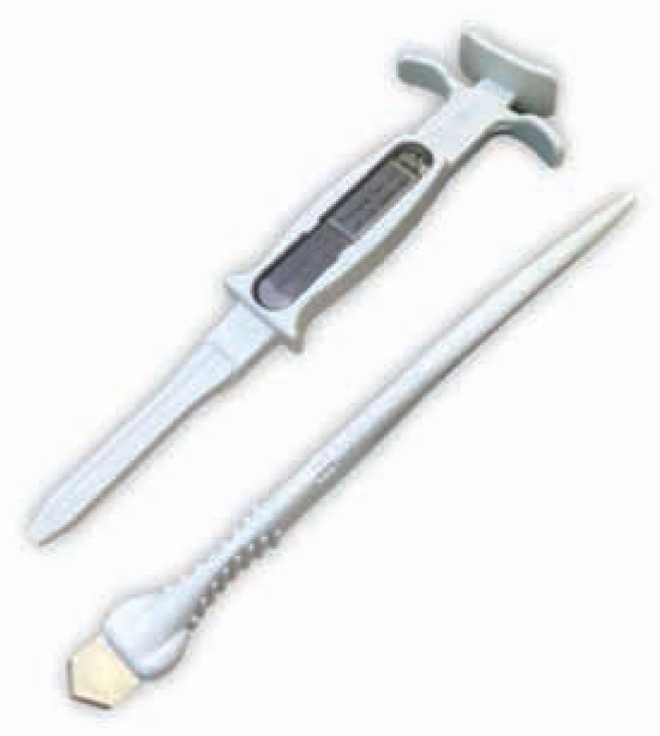
The insertion tool (top) and the incision tool (bottom) for the Confirm RX™ insertable cardiac monitor (Abbott Laboratories, Chicago, IL, USA).

The patient was sedated with 60 mg of propofol and the chest was prepped in the usual sterile fashion. The insertion site was marked at the fourth intercostal space, 0.5 cm to the left of the sternum at a 45-degree (°) angle. The insertion site was injected with 6 mL of 0.25% bupivacaine and the patient was given 1 g of prophylactic cefazolin prior to implantation. Using an incision tool **(Figure 1)**, an incision was made while applying slight retraction to the patient’s skin. Following the incision, the insertion tool, loaded with the monitor **(Figure 1)**, was inserted into the skin through the incision site at a 45° angle to the skin. The insertion tool was then moved parallel to the skin to ensure its placement parallel to the chest wall and advanced until the flared edges of the tool met the incision. While holding the insertion tool in place, the plunger was retracted until the monitor fell into the insertion channel. Then, using the plunger, the insertable cardiac monitor was advanced into the subcutaneous tissue. The insertion tool was subsequently removed, leaving the device in place.^2^

**Figure 2:**
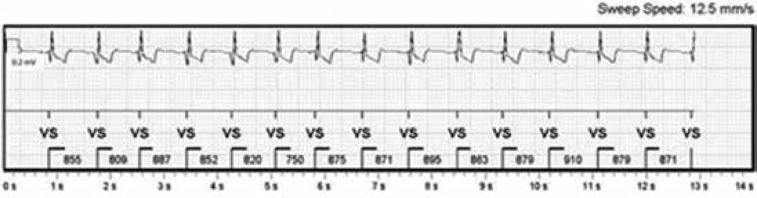
The initial transmission performed using the myMerlin™ application (Pacesetter Inc., Sylmar, CA, USA).

Following insertion, the monitor was tested and the R-wave signal amplitude was found to be 0.94 mV, with clear P-waves visualized on the tracings from the device. The incision site was closed with a single, simple interrupted suture and then covered with an absorbent island dressing. Following implantation, using the myMerlin™ application (Pacesetter Inc., Sylmar, CA, USA) on the patient’s smartphone, initial Bluetooth™ transmissions were made **(Figure 2)**, showing consistent tracings with the programmer. A subsequent follow-up was arranged based on the patient’s symptoms and transmissions. Thus far, remote transmissions have not shown any dysrhythmias.

## Conclusion

This case report shows the ability to obtain immediate rhythm information from a pediatric patient using a Bluetooth™-enabled device after a minimally invasive procedure. Currently, the vast majority of the available implantable cardiac monitoring systems involve a separate transmission unit to interrogate the device and report episodes to the managing physician. This often leads to inconsistencies and delays in reporting episodes. With the increasing use of smartphones among the younger generation, this technology will be particularly useful in making it easier to report episodes. It will also decrease the time from patient symptom occurrence to physician notification, since the notification and rhythm analysis can be performed almost immediately and without the need for any other device or interrogation system.
